# Determination of anticoagulant rodenticides in faeces of exposed dogs and in a healthy dog population

**DOI:** 10.1186/s13028-020-00531-5

**Published:** 2020-06-16

**Authors:** Kristin Opdal Seljetun, Vigdis Vindenes, Elisabeth Leere Øiestad, Gerd-Wenche Brochmann, Elin Eliassen, Lars Moe

**Affiliations:** 1 Department of Companion Animal Clinical Sciences, Faculty of Veterinary Medicine, Norwegian University of Life Sciences (NMBU), P. O. Box 369, Sentrum, 0102 Oslo, Norway; 2 Norwegian Poisons Information Centre, Norwegian Institute of Public Health, P. O. Box 222, Skøyen, 0213 Oslo, Norway; 3 Department of Forensic Sciences, Division of Laboratory Medicine, Oslo University Hospital, P. O. Box 4450, Nydalen, 0424 Oslo, Norway; 4 Institute of Clinical Medicine, Faculty of Medicine, University of Oslo, P. O. Box 1171, Blindern, 0318 Oslo, Norway; 5 School of Pharmacy, University of Oslo, P. O. Box 1068, Blindern, 0316 Oslo, Norway

**Keywords:** Brodifacoum, Bromadiolone, Canine, Difenacoum, Intoxication, Non-target animal, Rodenticide

## Abstract

**Background:**

Exposure to anticoagulant rodenticides (ARs) in dogs is among the most common causes of poisoning in small animal practice, but information about toxicokinetic of these rodenticides in dogs is lacking. We analysed blood and faeces from five accidentally exposed dogs and 110 healthy dogs by reversed phase ultra-high performance liquid chromatography-tandem mass spectrometry. The aim of the study was to estimate elimination of brodifacoum, bromadiolone and difenacoum after acute exposure, calculate the half-lives of these rodenticides in dogs, estimate faecal elimination in a litter of puppies born, and further to identify the extent of AR exposure in a healthy dog population.

**Results:**

Three dogs were included after single ingestions of brodifacoum; two dogs ingested bromadiolone and one dog ingested difenacoum. Maximum concentrations in faeces were found after day 2–3 for all ARs. The distribution half-lives were 1–10 days for brodifacoum, 1–2 days for bromadiolone and 10 days for difenacoum. Brodifacoum and difenacoum had estimated terminal half-lives of 200–330 days and 190 days, respectively. In contrast, bromadiolone had an estimated terminal half-life of 30 days. No clinical signs of poisoning or coagulopathy were observed in terminal elimination period. In blood, the terminal half-life of brodifacoum was estimated to 8 days. Faeces from a litter of puppies born from one of the poisoned dogs were examined, and measurable concentrations of brodifacoum were detected in all samples for at least 28 days after parturition. A cross-sectional study of 110 healthy domestic dogs was performed to estimate ARs exposure in a dog population. Difenacoum was detected in faeces of one dog. Blood and faecal samples from the remaining dogs were negative for all ARs.

**Conclusions:**

Based on the limited pharmacokinetic data from these dogs, our results suggest that ARs have a biphasic elimination in faeces using a two-compartment elimination kinetics model. We have shown that faecal analysis is suitable and reliable for the assessment of ARs exposure in dogs and a tool for estimating the AR half-lives. Half-lives of ARs could be a valuable indicator in the exposed dogs and provides important information for veterinarians monitoring AR exposure and assessment of treatment length in dogs.

## Background

Ingestion of anticoagulant rodenticides (ARs) is among the most common causes of poisoning in dogs worldwide [1, 2]. After ingestion, the ARs exert their effect by inhibiting vitamin K_1_ epoxide reductase. Consequently, regeneration of active vitamin K_1_ and formation of vitamin K_1_ dependent clotting factors II, VII, IX and X are disrupted [3]. The anticoagulant effect is mostly due to reduction of factors II and X, with plasma half-lives of 41 and 16.5 h, respectively [4]. After about 3–5 days, representing minimum two half-lives of factor II, circulating clotting factors are depleted and coagulopathy occur. The clinical signs and findings of ARs poisoning are unspecific and dogs may present with lethargy, pallor, dyspnoea, tachycardia and inappetence [5]. Prolonged prothrombin time (PT) and activated partial thromboplastin time (aPTT) confirms coagulopathy after ARs exposure.

The ARs are highly lipid soluble and accumulate in the liver [6]. In rat studies, liver elimination is suggested to be biphasic with a rapid distribution phase followed by a prolonged terminal phase [7, 8]. Information about liver elimination of ARs in dogs is lacking. ARs have an enterohepatic circulation which result in long half-lives and prolonged duration of anticoagulant effect [9]. Vitamin K_1_ is used as antidote in these poisonings, and treatment up to 4 weeks may be necessary [10]. The main elimination is through faeces, and faecal residues appears to be equivalent to hepatic residues for most ARs [8, 11]. Studies have demonstrated that ARs are detectable for a longer period in faeces compared to blood, indicating faecal analysis as a feasible method to determine half-lives of ARs in poisoned dogs [12–14].

Despite the frequency of ARs poisoning in dogs, reports with serial measurements of ARs concentrations, toxicokinetic data and estimated half-lives are scarce [14–16]. The primary aim of this study was to estimate the faecal elimination phase after a single ingestion and calculate the half-lives of ARs in acutely exposed dogs. The second aim was to estimate faecal elimination in a litter of puppies born from a previous poisoned dog. Finally, we aimed to estimate the occurrence of ARs exposure in a healthy dog population.

## Methods

### Animals

#### Exposed dogs

Six privately owned dogs shortly after a witnessed ingestion of ARs or with clinical signs of ARs poisoning brought to the University Small Animal Hospital at the Norwegian University of Life Sciences (NMBU) were included in the study (Table 1). Two dogs (cases 1 and 2) arrived at the NMBU displaying clinical signs of ARs poisoning. Cases 3–6 were examined 0.5–1.5 h after a witnessed rodenticide ingestion before occurrence of clinical signs. The amount ingested ARs were unknown in all cases. Patient demographics (including age, breed, sex, weight), information on residence, concurrent medications and previous possible exposures were recorded for all dogs.

**Table 1:** Characteristics and clinical presentation for six dogs exposed to anticoagulant rodenticides (ARs)

Case number	Age (years)	Weight (kg)	Clinical signs	Coagulation status	Detected ARs	Treatment	Duration of detectable AR concentrations after ingestion	
							Blood	Faeces
1	0.5	7	L, PMM, TP, DP, T	Prolonged	Brodifacoum	Vitamin K_1_, oxygen, fluids	7	At least 969^a^
2	2	6	L, PMM, TP, DP, T, I, LT	Prolonged	Brodifacoum	Blood transfusion, Vitamin K_1_, oxygen, fluids	53^b^	At least 894^b^
3	8	16	None	Normal	Brodifacoum	Emeticum, activated charcoal	9	At least 700
4	0.6	11	None	Normal	Bromadiolone	Emeticum, activated charcoal	n.d.	151
5	0.8	22	None	Normal	Bromadiolone	Emeticum, activated charcoal, fluids	n.d.	3
6	9.5	26	None	Prolonged (day 3)	Difenacoum	Emeticum, Vitamin K_1_	9	At least 653

*n.d.* Not detected, *L* lethargy, *PMM* pale mucous membranes, *TP* tachypnea, *DP* dyspnea, *T* tachycardia, *I* inappetence, *LT* low temperature

^a^Probable new exposure detected day 1032

^b^After first visit, day of ingestion unknown

Case 1 gave birth to four healthy, full-term puppies 1127 days after the first exposure and 95 days after a second suspected exposure. The puppies were included in the study for 12 weeks.

#### Healthy dogs

In this cross-sectional study of non-randomly selected 110 privately owned dogs were enrolled, selected at routine visits to veterinary clinics and national dog shows. Dogs were included from all 18 counties in Norway with a variety of living conditions (rural, suburban and urban) between November 2017 and October 2018. The dogs were of 59 different breeds, average age 5.2 years (range 1.5–13 years), average body weight 21 kg (range 2.9–70 kg) and both sexes (46 males and 64 females). According to owner’s signed declaration and information obtained in a comprehensive questionnaire, the dogs were healthy with no previous known exposure to ARs.

### Coagulation analyses

Blood samples for coagulation analyses (PT and aPTT) were obtained from the exposed dogs at each visit. Blood was collected into vacutainer tubes containing sodium-citrate (3.2%) and analysed by a Coag Dx Analyzer (IDEXX Laboratories Europe B.V., The Netherlands) within 2 h of collection. Elevated values day 3–5 after ingestion corresponded with the clinical signs in case 1. Coagulation was longer than the range of the method in case 2 at arrival. Both dogs received vitamin K_1_ and symptomatic treatment, and coagulation normalised. Vitamin K_1_ was administered per os for 50 days and 26 days in cases 1 and 2, respectively. Case 6 displayed increased PT (24 s; reference value 11–17 s) with normal aPTT and no clinical signs of poisoning at day 2 after ingestion. The dog was started on vitamin K_1_ treatment and coagulation normalised. Vitamin K_1_ therapy was continued for 26 days. The remaining dogs (cases 3, 4 and 5) displayed normal coagulation throughout the study.

### Sample collection

#### Exposed dogs

Blood and faecal samples were collected daily to weekly in the first month after exposure, followed by once a month until ARs were no longer detectable or the study ended. Faecal samples were collected by the owners after spontaneous defecation and brought to NMBU on the same day as blood was sampled. Faeces was collected in dark plastic bags, maintained at – 20 °C and within a few weeks lyophilized to dryness. Blood was collected in vacuum tubes containing sodium fluoride as preservative and potassium oxalate as anticoagulant. Blood samples were frozen (− 20 °C) shortly after collection and maintained frozen until analyses.

The litter of puppies was included from birth, and faecal samples were collected at day 1, 19, 23, 24, 27, 28 and 86 after parturition. Faeces was collected by the owner in dark plastic bags and maintained at – 20 °C. Within a few weeks the samples were brought to NMBU and lyophilized to dryness.

#### Healthy dogs

Blood and faeces were sampled once from each dog on the same day. Faeces was collected after spontaneous defaecation in dark plastic bags by the owner and brought to the veterinarian. Faeces was maintained at – 20 °C and were lyophilized to dryness within a few weeks after collection. Blood was collected in vacuum tubes containing ethylenediamine tetraacetic acid (EDTA) as anticoagulant. Blood samples were frozen (− 20 °C) shortly after collection and maintained frozen until analyses.

### Sample analyses

Blood and faeces were analysed for ARs at the Department of Forensic Sciences at Oslo University Hospital. Brodifacoum, bromadiolone, coumatetralyl, difenacoum, difethialone, and flocoumafen were analysed in this study, as all were previously registered for use in Norway. Sample preparation and faecal extraction were conducted as previously described [14]. Briefly, faecal samples were subjected to a liquid–liquid extraction with acetonitrile and dichloromethane, blood samples with ethyl acetate/heptane mixture. Separation followed by Waters Acquity reversed phase ultra-high performance liquid chromatography BEH C18 column (Waters Corporation, Milford, MA, USA) with a mobile phase consisting of 5 mM ammonium formate buffer (pH 10.2) and methanol. Positive electrospray ionization MS/MS detection was performed on a triple quadrupole mass spectrometer (Waters Corporation), using two multiple reaction monitoring transitions. An internal standard, warfarin d5, was added and analysed for all samples.

The extraction recoveries have previously been reported [14], and were 48% and 26% for difenacoum, 65% and 32% for bromadiolone, and 70% and 2% for brodifacoum, from blood and faeces respectively. Dog faeces is inhomogeneous with large variability in sample aliquot content. Even though large visible plant material, etc. were removed before sample preparation, this can influence both inter- and intra-individual extraction recovery. Good linearity and as well as precision and accuracy within ± 20% for all compounds were however found. In cases with more than one faecal sample per day, a mean of the ARs concentrations was calculated. Limits of quantification were 2.2 ng/g for difenacoum and 2.6 ng/g for brodifacoum and bromadiolone.

### Calculation of half-lives

The concentration versus time profile in the post-peak phase for drugs with first-order kinetics is an exponential function (dc/dt = – kC), where k is the elimination rate constant [17]. The elimination half-life (t_½_) can be calculate directly from the rate constant; t_½_ = ln 2/k or t_½_ = 0.693/k.

## Results

Characteristics, clinical presentation and treatment for the six dogs exposed to anticoagulant rodenticides are given in Table 1.

### Anticoagulant rodenticides analyses

In the dogs where faeces were collected during the first four days after ingestion (n=4), analyses displayed maximum ARs concentration at day 2–3 for bromadiolone, brodifacoum and difenacoum.

**Fig. 1:**
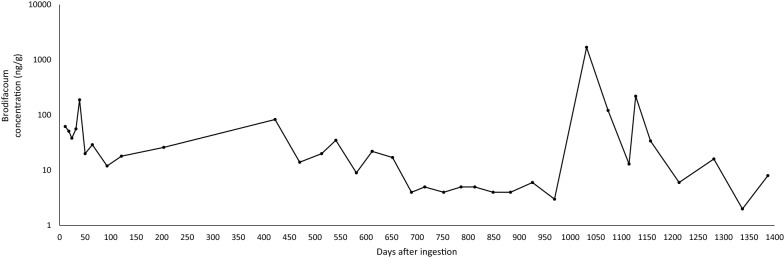
Logarithmic elimination kinetics of brodifacoum in faeces in case 1 (day 11–1387 after ingestion). The dog gave birth to four puppies day 1127 days after the first poisoning. Brodifacoum was detected for at least 969 days after first exposure, with an estimated terminal half-life of 330 days

**Fig. 2:**
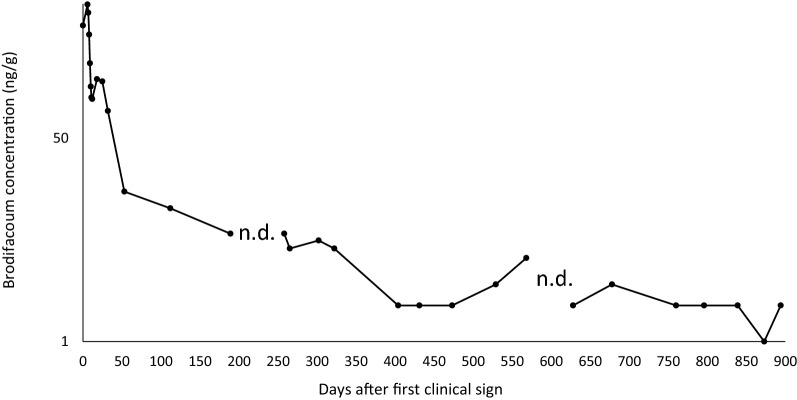
Logarithmic elimination kinetics of brodifacoum in faeces in case 2 (day 0–894 after first visit). Terminal elimination half-life was calculated to 200 days

**Fig. 3:**
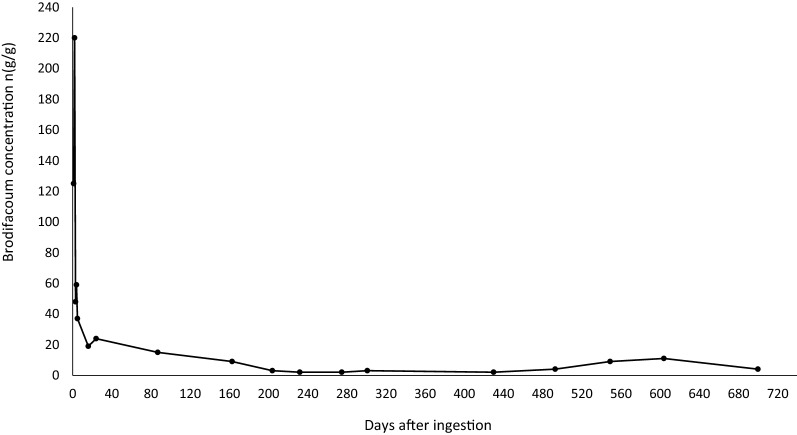
Elimination kinetics of brodifacoum in faeces in case 3 (day 1–700 after ingestion). Terminal elimination half-life was calculated to 300 days

Brodifacoum was identified in samples from three dogs (cases 1–3). In blood, brodifacoum was quantifiable for 7, 9 and 53 days (Table 1). For the remainder of the study, brodifacoum was identified in only trace amounts or not detectable as previously reported [14]. We identified low residues of brodifacoum in faeces of one dog for 969 days (Fig. 1). At day 1032 our results displayed a second peak in both blood and faecal levels, and a recent minor ingestion unknown to the owner was suspected. As the dog remained healthy with no recurrence of clinical signs, no vitamin K_1_ treatment was initiated. In case 2, brodifacoum was detected in faeces throughout the study and still detectable at the conclusion of the study (894 days; Fig. 2). Case 3 was lost to follow up after 700 days with still detectable levels of brodifacoum in faeces (Fig. 3).

In the litter of puppies born from case 1, low faecal concentrations of brodifacoum were detected in all samples up to 28 days after parturition, but not detected at day 86.

**Fig. 4:**
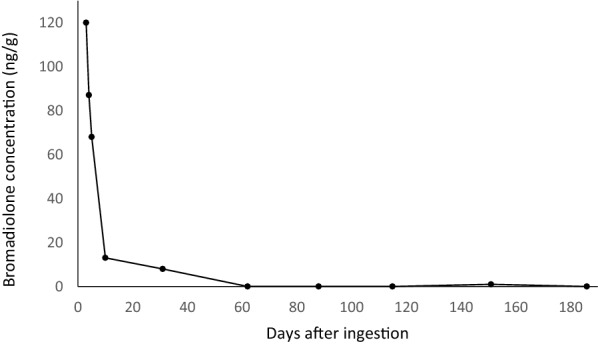
Elimination kinetics of bromadiolone in faeces in case 4 (day 3–186 after ingestion) Terminal elimination half-life was calculated to 30 days

**Fig. 5:**
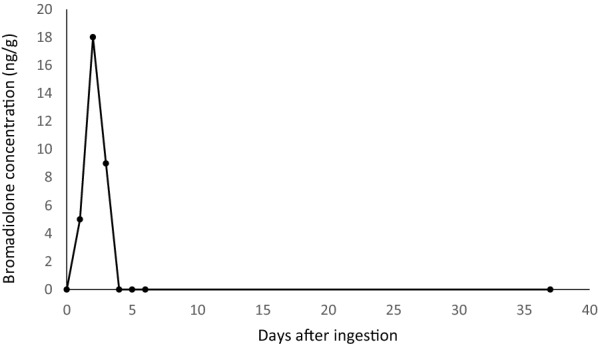
Elimination kinetics of bromadiolone in faeces in case 5 (day 0–37 after ingestion)

Bromadiolone was detected in two dogs in the study (cases 4–5). Both dogs remained healthy throughout the study period with no clinical signs of ARs exposure. Coagulation remained normal and bromadiolone was not detected in blood. In faeces, bromadiolone was identified for 151 days in case 4, however samples were negative from day 62 to 115 (Fig. 4). In case 5, bromadiolone was detected for 3 days (Fig. 5).

**Fig. 6:**
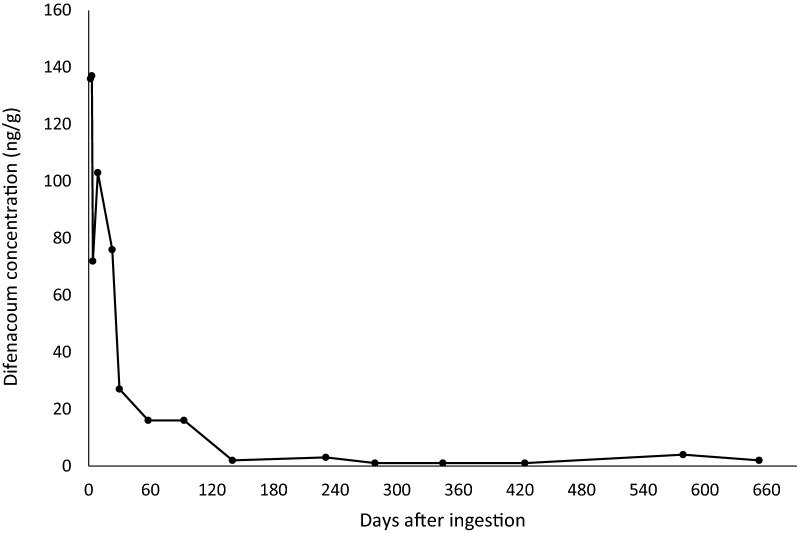
Elimination kinetics of difenacoum in faeces in case 6 (day 2–653 after ingestion) Terminal elimination half-life was calculated to 190 days

Difenacoum was only identified in case 6. In blood, difenacoum was detected in trace amounts until day 9. Throughout the remainder of the study, difenacoum was identified in trace amounts in blood or not detected. In faeces, difenacoum was detected throughout the study and still detectable at the conclusion of the study (653 days; Fig. 6).

### Estimated half-lives

In faeces, the serial levels indicate biphasic elimination and a two-compartment model for all ARs in dogs. The distribution half-life varied between the substances with 1–10 days in brodifacoum (case 1–3), 1–2 days in bromadiolone (case 4–5) and 10 days in difenacoum (case 6). The terminal half-lives were prolonged in brodifacoum with 200–330 days and difenacoum with 190 days. Elimination phase of bromadiolone was estimated in case 4 only, due to low concentration in case 5, and terminal half-life was calculated to 30 days.

In blood, as a result of low concentrations, distribution and elimination half-lives were only possible to estimate in case 2. Serum distribution half-life of brodifacoum was calculated to 1 day and the terminal half-life to 8 days.

### The healthy dog population

Faeces and blood from 110 healthy domestic dogs assumed previously unexposed to ARs were sampled to establish prevalence of AR in a healthy dog population. The analyses revealed difenacoum in faeces (2 ng/g) of only one dog. All blood samples were negative for brodifacoum, bromadiolone, coumatetralyl, difenacoum, difethialone, and flocoumafen.

## Discussion

This study confirms a biphasic elimination of brodifacoum, difenacoum and bromadiolone in faeces of dogs. Our findings suggest that ARs in dogs are stored for months in different tissues in the body after a single ingestion.

We estimated plasma terminal half-life of brodifacoum to 8 days in one case. A median plasma half-life of brodifacoum was estimated to 2.4 days (range 0.9–4.7 days) in analyses of seven poisoned dogs by high performance liquid chromatography (HPLC) in 1997 [16]. An experiment in four dogs in 1992 with repeated ingestions for three consecutive days estimated the serum half-life by HPLC to 6 ± 4 days [15]. As our result is based on only one case, comparisons are uncertain, but the prolonged half-life in our case could be due to improved analytical methods over the past 20 years.

Our study demonstrates that faecal sampling is a feasible method of monitoring ARs exposure in dogs, although faeces display a large variability in extraction recovery due to inhomogeneous samples and presence of plant material. We detected a substantially longer persistence of ARs in faeces compared to blood, corresponding to similar differences between liver and blood in quail and possums [18, 19].

Brodifacoum displayed a distribution phase of 1–10 days in faeces. The variation between the cases is probably partly due to exposure to different amounts and more frequent sampling might have improved the estimated distribution phase. However, due to poor clinical condition in two of the dogs, defecation was sparse during the first days after presentation. In addition, the amount distributed is influenced by initial decontamination of gastric emptying and activated charcoal which differed between the dogs. Although case 3 displayed no clinical signs of exposure, coagulation remained normal and no treatment was administered after initial decontamination, brodifacoum was still detectable for more than 700 days in faeces. Brodifacoum was detected in faeces of cases 1 and 2 for 894–969 days until the end of the study. Faecal terminal half-lives in the three dogs were estimated to 200–330 days. A previous investigation demonstrated a variation in individual susceptibility to brodifacoum [20], and this could contribute to the differences between the dogs detected in our study. In addition, as brodifacoum was present in faeces in all dogs at drop-out or conclusion of the study, different estimated terminal half-lives of these dogs are conceivable. As no studies have examined half-lives of brodifacoum in dogs, comparisons to other species have been done. A half-life of 307 days in liver was observed in mice after a single ingestion of brodifacoum, which corresponds with our findings [21]. In sheep, brodifacoum was below the limit of detection in faeces at day 32 after a single ingestion [13]. The discrepancy from our study could be due to different analytical methods. Secondly, difference in level of detection is a contributing factor, with 0.05 mg/kg (equivalent to 50 ng/g) in the sheep compared to our limit of quantification of 2.6 ng/g. In addition, difference in species could contribute to the variance as metabolism and inhibition of vitamin K 2,3-epoxide reductase of ARs vary between species [22].

Bromadiolone was less persistent in faeces compared to brodifacoum in our study, correlating to previous studies with a longer hepatic persistence of brodifacoum compared to bromadiolone both in mice and rats [20, 21]. Bromadiolone displayed a biphasic elimination in faeces with an initial distribution phase of 1–2 days, equivalent to an experiment in pigs [23]. We calculated faecal terminal half-life of bromadiolone to 30 days in case 4. This correlates to an estimated liver half-life of 28 days and 24 days after a single ingestion in mice and humans, respectively [21, 24]. Our estimate is however based on only one case.

There are few reports estimating half-lives of difenacoum. A half-life of 62 days in liver was detected after a single ingestion in mice [21]. Several experiments have been conducted in rats, and a half-life of 120–128 days in liver has been suggested [7, 25]. However, in contrast to dogs, rats lack gall bladder and continuously secrete bile, problematizing direct comparisons between these species [26]. Difenacoum was detected in faeces in case 6 when the study ended 653 days after ingestion, and the estimated half-life was 190 days. Our estimate is however only based on the findings from one dog exposed to difenacoum. The prolonged elimination in this dog differs from our findings of shorter terminal elimination of bromadiolone but corresponds to the detected elimination of brodifacoum in cases 1–3. Corresponding pharmacokinetics of difenacoum and brodifacoum were detected in an experiment with analyses of plasma concentrations in rabbits [27].

In the 110 healthy dogs assumed unexposed to ARs, we detected one dog with low faecal concentrations of difenacoum. A previous investigation of 115 domestic pets revealed two dogs with trace amounts of diphacinone in the liver [28]. However, clinical status of the dogs was not specified in the study. Due to the limited number of dogs, where ARs were found, detection of predisposing factors contributing to ARs exposure was not possible. More research is needed to detect the cause and occurrence of ARs exposure in the healthy dog population.

The dogs did not display any clinical effects of their subtoxic AR concentrations in the months after exposure. Previous studies have detected reduced body condition of wildlife with sublethal ARs concentrations [29–31]. In addition, subtoxic ARs levels are suspected to increase susceptibility to pathogens, while others have not found such association [32–34]. Several studies have examined AR-related effects on the immune system in different species, and ARs changed the expression of immune-related genes in bobcats (*Lynx rufus*), increased levels of immature red blood cells in red-tailed hawks (*Buteo jamaicensis*), and decreased production of cytokines in domestic cats [34–36]. Further studies are required to assess the relevance in dogs with sublethal ARs concentrations.

Earlier studies have detected teratogenic effects, abortion and postpartum death after ARs exposure in pregnant animals [37, 38]. Stillbirth and neonatal death were seen in one report at least 4 weeks after a possible brodifacoum exposure of an unaffected bitch not displaying clinical signs of coagulopathy [39]. However, in the present study, case 1 gave birth to four full-term, healthy puppies after exposure. Brodifacoum was detected in faeces in both puppies and bitch for 1 month after birth, but all remained asymptomatic.

## Conclusions

Faecal analysis has shown to be suitable and reliable for the assessment of ARs exposure in dogs and a valuable tool in estimating ARs half-lives in dogs. This study suggests that brodifacoum and difenacoum might be present in dogs’ faeces after a single ingestion for more than 700 days and 650 days, respectively, and were still detectable at the conclusion of the study. Bromadiolone showed a comparatively shorter half-life in dogs. In a litter of puppies born from a poisoned dog, low faecal concentrations of ARs were detected for at least 28 days after parturition. The results may indicate a rather low prevalence of AR exposure among healthy dogs in Norway, but due to the limited number of dogs in this study, detection of prevalence and predisposing factors contributing to ARs exposure were not possible.

## Data Availability

The datasets used and/or analysed during the current study are available from the corresponding author on reasonable request.

## Abbreviations

aPTT: Activated partial thromboplastin time
ARs: Anticoagulant rodenticides
HPLC: High performance liquid chromatography
NMBU: Norwegian University of Life Sciences
PT: Prothrombin time
